# The citation of relevant systematic reviews and randomised trials in published reports of trial protocols

**DOI:** 10.1186/s13063-016-1713-6

**Published:** 2016-12-07

**Authors:** Nikolaos Pandis, Padhraig S. Fleming, Despina Koletsi, Sally Hopewell

**Affiliations:** 1 Department of Orthodontics and Dentofacial Orthopedics, School of Dental Medicine, Medical Faculty, University of Bern, Bern, 3010 Switzerland; 2 Barts and The London School of Medicine and Dentistry, Queen Mary University of London, London, UK; 3 London School of Hygiene and Tropical Medicine, University of London, London, UK; 4 Nuffield Department of Orthopaedics, Rheumatology and Musculoskeletal Sciences, Oxford Clinical Trials Research Unit, University of Oxford, Oxford, UK

**Keywords:** Randomised trials, Systematic reviews, Citation, Protocol, SPIRIT statement

## Abstract

**Background:**

It is important that planned randomised trials are justified and placed in the context of the available evidence. The SPIRIT guidelines for reporting clinical trial protocols recommend that a recent and relevant systematic review should be included. The aim of this study was to assess the use of the existing evidence in order to justify trial conduct.

**Methods:**

Protocols of randomised trials published over a 1-month period (December 2015) indexed in PubMed were obtained. Data on trial characteristics relating to location, design, funding, conflict of interest and type of evidence included for trial justification was extracted in duplicate and independently by two investigators. The frequency of citation of previous research including relevant systematic reviews and randomised trials was assessed.

**Results:**

Overall, 101 protocols for RCTs were identified. Most proposed trials were parallel-group (*n* = 74; 73.3%). Reference to an earlier systematic review with additional randomised trials was found in 9.9% (*n* = 10) of protocols and without additional trials in 30.7% (*n* = 31), while reference was made to randomised trials in isolation in 21.8% (*n* = 22). Explicit justification for the proposed randomised trial on the basis of being the first to address the research question was made in 17.8% (*n* = 18) of protocols. A randomised controlled trial was not cited in 10.9% (95% CI: 5.6, 18.7) (*n* = 11), while in 8.9% (95% CI: 4.2, 16.2) (*n* = 9) of the protocols a systematic review was cited but did not inform trial design.

**Conclusions:**

A relatively high percentage of protocols of randomised trials involves prior citation of randomised trials, systematic reviews or both. However, improvements are required to ensure that it is explicit that clinical trials are justified and shaped by contemporary best evidence.

**Electronic supplementary material:**

The online version of this article (doi:10.1186/s13063-016-1713-6) contains supplementary material, which is available to authorized users.

## Background

The onus on clear research reporting and indeed optimal yield from randomised trials has been highlighted increasingly in recent years with much research now accepted as being suboptimal with ensuing financial and systemic waste [1]. Among the more pressing shortcomings are failure to consider questions of relevance to clinicians and patients, inappropriate design and methods, publication bias, and biased and incomplete reporting [2].

Accepted prerequisites for randomised trials are the existence of genuine uncertainty concerning the relative merits of competing interventions and appropriate design to permit meaningful answers [3]. An appreciation of the existence of previous research is therefore necessary to avoid unnecessary duplication, and due consideration of the evidence base is important in informing appropriate design and methodology. Notwithstanding this, replication of previous studies may be justified in confirming previous results or in an effort to assess the generalizability of novel findings [4].

It is accepted that relevant research, including systematic reviews where they exist and randomised trials, should be cited in the introduction section of reports of randomised controlled trials (RCTs) [3]. There are also growing concerns in relation to publication bias and selective outcome reporting in biomedical journals [5, 6]. Consequently, in recent years there has been an increasing drive to publish research protocols both to prevent unwanted duplication and to mitigate the risk of reporting bias [7]. The Standard Protocol Items: Recommendations for Interventional Trials (SPIRIT) guidelines [7] aim to improve the reporting and quality of clinical trial protocols and incorporate a checklist of recommended items to include in the trial protocol [7]. Within the SPIRIT guidelines, citation of prior research in the introduction section and justification of randomised trials on the basis of gaps in the underlying evidence base are advocated [7]. The SPIRIT checklist strongly recommends placing: “The trial in the context of available evidence, it is strongly recommended that an up-to-date systematic review of relevant studies be summarised and cited in the protocol.”

Previous research has addressed the issue of recognition of prior studies in stimulating or informing further research [8–10]. Robinson and Goodman [11] in an analysis of RCTs contributing to meta-analyses identified that less than one quarter of relevant trials had been cited. Similarly, Clarke and Hopewell [10] in a survey of five leading medical journals reported sparse referencing of systematic reviews either in the introduction or discussion sections. Consequently, randomised trial reports may routinely fail to place research findings in appropriate context potentially hampering the end user’s ability to reach balanced, informed decisions about important healthcare interventions. Failure to consider the available and latest evidence may jeopardize the justification for new randomised research on ethical grounds.

To our knowledge no studies exist assessing the justification for randomised trials based on the available evidence at the protocol stage and in accordance with SPIRIT. The aim of this meta-epidemiological study was to assess the extent to which published protocols of randomised trials adhere to SPIRIT and include an updated systematic review to inform the trial design in the background or rationale sections.

## Methods

### Sample

All published protocols of planned randomised controlled trials (RCTs) were identified over a 1-month period (December 2015) indexed in PubMed based on a defined search strategy (Table 1).

**Table 1:** Search strategy for protocol selection

“protocol[ti] AND random*”
Filter for “randomised trials” and time range
1. randomised controlled trial [pt] OR controlled randomised trial [pt] OR randomised [tiab] OR randomised [tiab] OR placebo [tiab] OR randomly [tiab] OR trial [tiab]
2. protocol [tiab]
3. systematic review OR meta-analysis [ti] OR meta analysis [ti] OR review [ti] OR Review [pt] OR Meta-Analysis [pt] OR Comment [pt] OR Letter [pt] OR Editorial [pt] OR News [pt]
4. #1 AND #2 NOT #3

### Data extraction

Titles and abstracts were screened by one author (NP) in order to select all eligible publications. The selected citations were entered in Endnote reference management software and the corresponding full texts were retrieved for further evaluation. The criteria for defining a report as a “randomised controlled trial protocol” were as follows:

- Document describing the objectives, design, methodology, statistical considerations, and organisation of a clinical trial including justification and rationale for the trial.
- Cochrane definition of a trial as randomised: “the individuals (or other units) followed in the trial were assigned prospectively to one of two (or more) alternative forms of health care using random allocation.”

The following information was extracted in duplicate by two authors (NP, PSF) from each eligible protocol: title, first author name, number of authors, country and geographic region (i.e. Europe, Americas, Asia, Other) of first author, details of registration, funding, number of trial sites, trial design, funding type, conflict of interest, and data sharing. Following the SPIRIT recommendation within the introduction section the research context used to inform or justify the trial was assessed in one of six ways [12]:

- randomised trial is the first to address the question
- updated systematic review was used to inform trial design
- systematic review and new trials (published since the systematic review was published) were used to inform trial design
- previous systematic review was discussed but was not used in trial design
- references to other randomised trials
- no references to other randomised trials or claim to be the first trial

### Data analysis

Data were independently extracted by two investigators and entered on pre-piloted standardized forms for the eligible protocols. Initial calibration was performed between the two researchers on ten articles. Disagreements were resolved by discussion or, if necessary, with adjudication by a third reviewer and a consensus was reached for all protocols. Descriptive statistics were undertaken and data were tabulated with respect to selected protocol characteristics with the use of STATA® version 14.1 software (Stata Corporation, College Station, TX, USA).

## Results

**Fig. 1:**
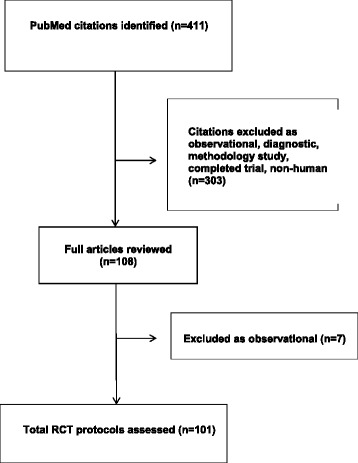
RCT protocol selection flow diagram

The PubMed search identified 411 records, 310 of which were excluded as they did not satisfy the inclusion criteria resulting in 101 protocols suitable for full data extraction (Fig. 1). The Additional file 1 includes the raw data extracted. A variety of medical conditions were considered in the selected protocols (Table 2). The majority of studies were undertaken in multiple centres (84.2%). In terms of geographic region, the highest percentage of studies was carried out in Europe (54.5%; Table 2). Most proposed trials were parallel-group (*n* = 74; 73.3%), while 9.9% (*n* = 10) were cluster designs. The vast majority of studies reported funding (94.1%) with non-industry funding in isolation in 82.2%. Conflict of interest statements were made in the vast majority of protocols (*n* = 96; 95.0%), with the majority having no conflicts to declare (*n* = 77; 76.2%). No commitment to data sharing was made in the majority of protocols (*n* = 93, 92.0%), 7% (*n* = 7) reported that data will be available upon request and only one (1%) protocol indicated the data repository.

**Table 2:** Distribution of demographic variables within the protocols assessed (*n* = 101)

Characteristics of assessed protocols	Total	
	N (101)	%
Journal		
Annals of Translational Medicine	1	1.0
BMC umbrella journals	23	22.7
BMJ Open	15	14.8
Clinical and Translational Allergy	1	1.0
Contemporary Clinical Trials	4	4.0
Danish Medical Journal	1	1.0
Implementation Science: IS	1	1.0
JAMA Surgery	1	1.0
JMIR Research Protocols	2	2.0
Journal of Advanced Nursing	3	3.0
Journal of Diabetes Science and Technology	1	1.0
Nutrients	1	1.0
Reproductive Health	1	1.0
Springer Plus	1	1.0
The Journal of Cardiovascular Nursing	1	1.0
Trials	44	43.5
Subject		
Behavioural	49	48.5
Biological or vaccine	1	1.0
Dietary supplement	6	5.9
Drugs	18	17.8
Surgery or procedure	14	13.9
Other	13	12.9
No. centres (according to authors’ affiliations)		
Multi-centre	85	84.2
Single-centre	16	15.8
Continent of authorship		
America	19	18.8
Europe	55	54.5
Asia and other	27	26.7
No. sites (based on trial conduct)		
Multiple	52	51.5
Single	49	48.5
Trial design		
Parallel	74	73.3
Cluster	10	9.9
Non-inferiority	5	5.0
Superiority	2	2.0
Other (or mixed-type designs)	10	9.8
Type of funding		
Non-industry	83	82.2
Part industry	5	4.0
Industry	7	6.9
None/unknown	6	5.9
Conflict of interest		
No	77	76.2
Yes	19	18.8
Not described	4	4.0
Unclear	1	1.0
Data sharing		
Data upon request	7	7.0
Data stored in central repository	1	1.0
No information provided	93	92.0
Total	101	100.0

In terms of citation of relevant research (Table 3), reference to a previous systematic review with additional randomised trials was found in 9.9% (95% CI: 4.9, 17.5) (*n* = 10) of protocols and without additional trials was alluded to in 30.7% (95% CI: 21.9, 40.7) (*n* = 31). A randomised controlled trial was not cited in 10.9% (95% CI: 5.6, 18.7) (*n* = 11), while in 8.9% (95% CI: 4.2, 16.2) (*n* = 9) of the protocols a systematic review was cited but did not inform trial design. Of the cited systematic reviews (*n* = 41), used to inform trial design, 41.5% (*n* = 17) were Cochrane reviews.

**Table 3:** Distribution of protocol characteristics in terms of citation of relevant research (*n* = 101)

Protocol characteristics	Total (*n* = 101)		
	N	%	95% CI
Claims that randomised trial is the first to address the question	18	17.8	10.9, 26.7
Contains an updated systematic review used to inform trial design	31	30.7	21.9, 40.7
Contains systematic review and new trials (published since the systematic review was published) that were used to inform trial design	10	9.9	4.9, 17.5
Previous systematic review discussed but not used in trial design	9	8.9	4.2, 16.2
Contains references to other randomised trials	22	21.8	14.2, 31.1
Does not contain references to other randomised trials or claim to be the first trial	11	10.9	5.6, 18.7

From the 101 included protocols 89 of those were published in journals endorsing the SPIRIT guidelines.

## Discussion

The CONSORT statement has stipulated that findings from a randomised trial should be placed in the context of the “totality of the available evidence” [3]. Similarly, when designing a new study it is important that the setting and methodology is informed by previous research. This cross-sectional analysis is the first to assess the citation of high-level research, including both systematic reviews and randomised controlled trials, in reports of published protocols of planned randomised trials. Overall, 41% of the protocols cited a systematic review or a randomised trial that was used to inform trial design.

Previous analyses of reports of randomised trials have indicated that systematic reviews are infrequently cited in reports of randomised trials with sequential audits of trials published in leading medical journals alluding to failure to refer to systematic reviews in 24% of reports in 1997, 10% in 2001, 33% in 2005, 46% in 2009 and 39% in 2012 [8–10]. This finding has arisen despite the pervasion of systematic reviews with in excess of 5000 full Cochrane reviews now published in the Cochrane Database of Systematic Reviews [13] and several thousand more systematic reviews published on an annual basis [14]. Our study indicates that trial justification based on systematic reviews and randomised trials at the protocol level is slightly higher compared to published trials and this finding is encouraging, despite the potential imprecision in obtaining these higher values reflected by the wide confidence intervals. Reasons for this are unclear at this stage but it could be speculated that the utility of systematic reviews has improved over time, that a greater onus on trial justification exists at the protocol stage with funding agencies placing an emphasis on evidence of a recent systematic review to justify further clinical research. However, this is the first study to assess this and further research may be required to confirm this pattern within research protocols.

The SPIRIT guidelines were developed in response to inadequate description of trial details such as outcomes and interventions, but also due to unclear description of trial methodology [7]. These shortcomings may prompt protocol amendments, might hamper trial conduct and can ultimately lead to inadequate reporting [15]. Moreover, it is important that previous research is considered at the design stages to avoid unnecessary duplication, to inform trial design in order to maximize the yield from expensive and lengthy randomised studies [1]. Where genuine uncertainty concerning the effectiveness of an intervention is lacking, undertaking such a trial is also considered unethical. It is therefore recommended both within the SPIRIT and CONSORT statements that relevant randomised trials and systematic reviews are identified. It is accepted, however, that compliance with established reporting and conduct guidelines is suboptimal throughout the biomedical literature [16]. While meta-epidemiological studies focusing on compliance with SPIRIT have not yet been reported, the present investigation does report some encouraging results.

A potential limitation of the present study is the possibility that there may not have been relevant trials or systematic reviews to cite at the time of the design of the new trial. It is therefore possible that the prevalence of failure to consider relevant research may be overstated slightly. However, previous studies have attempted to identify similar studies [11] but have found broadly similar rates of isolated reports. Furthermore, the present study was limited to a restricted time period and few sources. The search strategy looked specifically for protocols in the title and therefore it is possible that a number of trial protocols might have been overlooked. However, the journals included are likely to represent best practice in terms of protocol design and reporting as most of them endorse SPIRIT guidelines with the majority of trial protocols likely remaining unpublished at this juncture. It is therefore likely that the prevalence of failure to identify relevant related research found in the present study represents a best-case scenario. Finally, a limited number of pharmacological studies were identified over the study period; further research focusing on the protocol reporting characteristics for these studies, in particular, would therefore be welcome.

## Conclusions

A relatively high percentage of protocols of randomised trials involves prior citation of either randomised trials, systematic reviews or both. Overall, 41% of protocols involved citation of a systematic review or a randomised trial that was used to inform trial design.

## Additional file

Additional file 1: Data extraction file with raw data. (XLSX 31 kb)
